# Does education years delay Alzheimer’s disease in cognitively normal oldest old?

**DOI:** 10.1002/alz.089080

**Published:** 2025-01-09

**Authors:** Hamid R Sohrabi, Kathryn G Goozee, Michael Weinborn, Stephanie Rainey Smith, Kevin Taddei, Ralph N Martins

**Affiliations:** ^1^ Centre for Healthy Ageing, Health Future Institute, Murdoch University, Perth, Western Australia Australia; ^2^ Macquarie University, North Ryde Australia; ^3^ Australian Alzheimer’s Research Foundation, Perth, Western Australia Australia; ^4^ Edith Cowan University, Joondalup Australia; ^5^ KaRa Institute of Neurological Diseases, Sydney Australia; ^6^ Macquarie University, Macquarie Park, NSW Australia; ^7^ The University of Western Australia, Perth, Western Australia Australia; ^8^ Centre for Healthy Ageing, Murdoch University, Murdoch, Western Australia Australia; ^9^ Edith Cowan University, Perth, Western Australia Australia; ^10^ Murdoch University, Perth, Western Australia Australia; ^11^ Department of Biomedical Sciences, Macquarie University, Macquarie Park, NSW Australia

## Abstract

**Background:**

Research on cognitive reserve (CR) in individuals aged 80 years old and above has resulted in inconsistent findings, mostly showing a relationship with baseline cognitive abilities but not follow up assessments. The effects of amyloid burden on the relationship between CR, cognitive decline and dementia in oldest old warrants further study in the presence of APOE e4. We hypothesised that CR in oldest old (≥80 yrs old) adults will result in different trajectories, depending on being amyloid PET positive or negative. Specifically, this study was to determine whether the relationship between CR and cognition is mediated by amyloid load in very old individuals.

**Method:**

Data of 115 participants aged 80 years and above at baseline and one follow‐up was analysed for this study. Participants were recruited from the Western Australia Memory Study (WAMS, Perth) and the Kerr Anglican Retirement Village Initiative in Ageing Health (KARVIAH) Study (Sydney, Australia). In both sites participants have undergone amyloid imaging, as well as a comprehensive neuropsychological assessment, *APOE* genotyping and AD‐related blood‐based biomarkers analysis.

**Result:**

Preliminary results indicated a non‐significant relationship between education years, as the proxy for CR, and baseline cognition. Similar not‐significant findings was observed between CR and amyloid load. Individuals positive or negative for amyloid burden on the brain at baseline, as well as carriage of APOE e4 did not differ on years of education. Of interest, APOE e4 allele carriers were not different from not‐carriers on amyloid load in the brain. Due to nonsignificant relationships between the variables of interest, in both cross‐sectional as well as longitudinal data, further mediation analysis was not carried out.

**Conclusion:**

Our nonsignificant results indicated that the commonly reported relationship between CR, cognitive function, amyloid load in older adults may not be present in individuals aged 80 years old and above. Although these findings are consistent with previous reports, other factors including longevity genetic factors that promote preserving cognition in oldest adults as well as lifestyle factors should be considered. Further analysis of such factors is currently ongoing and may result in interesting outcomes for this cohort.

